# Interannual temperature rise leads to more uniform phenological matching between invasive *Stellera chamaejasme* and pollinators across elevations

**DOI:** 10.3389/fpls.2024.1445083

**Published:** 2024-10-11

**Authors:** Chenxin Miao, Jun Du, Wen Wang, Juanjuan Wu, Longqing Wu, Kehai Zhang, Xuee Ma

**Affiliations:** ^1^ Key Laboratory of Ecological Safety and Sustainable Development in Arid Lands, Northwest Institute of Eco-Environment and Resources, Chinese Academy of Sciences, Lanzhou, China; ^2^ University of Chinese Academy of Sciences, Beijing, China; ^3^ Academy of Water Resources Conservation Forests in Qilian Mountains of Gansu Province, Zhangye, China

**Keywords:** flowering phenology, potential pollinator, phenological matching, rising temperature, elevational pattern

## Abstract

Exploring how environmental changes induce alterations in the phenology matching between plants and pollinators is significant for predicting species’ reproductive output and population dynamics. Our study focused on the invasive poisonous weed *Stellera chamaejasme*, widely distributed in the Qilian Mountains, China. By continuously monitoring its flowering phenology and flower visitors’ activities across different elevational ranges, we compared phenological matching patterns between *S. chamaejasme* and its potential pollinators across years with varying environmental temperatures. We found that *S. chamaejasme*, a typical early-flowering alpine species, begins its flowering in early June. Despite variations in the composition of flower-visiting insects across elevations and years, it maintained stable interactions with four major groups: *Meloidae*, *Tachinidae*, *Scarabaeidae*, and *Noctuidae*. Phenological mismatches between the peak flowering period of *S. chamaejasme* and the peak abundance of major potential pollinators were generally observed across its range, with higher phenological matching at higher elevations. This enhanced matching at higher elevations may drive the rapid invasion of *S. chamaejasme* in these areas. In the year with higher ambient temperature, phenological matching increased across its range, and its elevational sensitivity decreased, potentially contributing to its ongoing expansion in different elevations. The results of our study advance a new insight into the population expansion of invasive species in mountain ecosystems.

## 1 Introduction

As a central focus of population ecology, the study of plant population dynamics has garnered widespread attention, with climatic conditions as one of the primary drivers (Tay et al., 2018). One of the critical challenges in predicting the impacts of climate change on organisms is understanding how environmental conditions influence the dynamics of population sizes and numbers (Tomasek et al., 2019). Although extensive research has explored the ecological and evolutionary processes influencing plant dispersal (such as local adaptation and physiological constraints), our understanding of the mechanisms underpinning plant responses to environmental changes, especially concerning reproductive dependency, still exhibits certain deficiencies (McLean et al., 2016; Oldfather and Ackerly, 2019). For seed plants, reproductive success dictates seed output, forming the foundation for population renewal and expansion. This process relies on mutualistic interactions between flowering plants and pollinating insects (Dai et al., 2018). This mutualism arises from long-term evolution and adaptation under specific phenological and spatiotemporal constraints, rather than by chance (Santamaría and Rodríguez-Gironés, 2007; Stang et al., 2009). A greater degree of phenological overlap is crucial for efficient reproduction (Visser and Gienapp, 2019).

Increasing global evidence indicates a trend of phenological shifts in biological systems (Root et al., 2003). Asynchronous phenological responses among species, resulting from differing reactions to climate change, have been particularly noted (Wang et al., 2024). Specifically, the differential sensitivities of plants and pollinators to environmental changes, especially climate change, have caused shifts in the timing of plant flowering and pollinator activity (Petanidou et al., 2014; Kharouba et al., 2018). These changes in phenological overlap periods vary among interacting species (Wang et al., 2024). For instance, plants that flower earlier due to warming temperatures often face a scarcity of pollinators, while some pollinators may experience periods of food shortage or absence, resulting in reduced plant seed set and a decline or disappearance of pollinating insects, thereby severely impacting community structure (Kudo and Ida, 2013). Conversely, some studies show that the activity times of insects and flowering times of plants may shift synchronously under climate warming, thus maintaining their mutualistic relationships and minimizing impacts on plant reproductive success (Rafferty and Ives, 2011; Forrest, 2015). Therefore, it is crucial to investigate how environmental changes alter the phenological matching between plants and pollinators (Li et al., 2024).

Compared to the latitudinal and longitudinal geographical boundaries of flora and fauna, the elevational distribution in mountainous systems represents a condensed spatial distribution pattern (Van Beusekom et al., 2015). Due to the highly heterogeneous habitat types within a relatively small spatial scale and their high sensitivity to climate change, mountainous regions provide a good platform for elucidating the dynamics of phenological matching in species interactions under climate change (Tito et al., 2020). This study focuses on the native invasive poisonous weed *Stellera chamaejasme*, widely distributed in the Qilian Mountains, and utilizes elevational gradients as a means of analysis to investigate the environmental responses of phenological matching between *S. chamaejasme* and its pollinators (Li et al., 2024). *S. chamaejasme* is self-incompatible and relies entirely on pollen vectors for seed production. As an indicator species of grassland degradation, the rapid spread of *S. chamaejasme* has altered herbaceous community composition and reduced grassland quality, negatively impacting the sustainable development and ecological security of grassland pastoralism (Li et al., 2019). Current explanations for the mechanisms behind the spread of *S. chamaejasme*, such as the “fertility island” effect and allelopathic inhibition, have primarily focused on the positive feedback effects of environmental selection pressure release on growth fitness from either the plant or soil perspective (Li et al., 2019). However, these explanations have yet to fully elucidate the rapid population spread (Van Beusekom et al., 2015). The study of the phenological matching between *S. chamaejasme* and its pollinators in the context of environmental change may provide an additional explanation for understanding population expansion. Therefore, our research aims to provide new evidence for understanding the population expansion of *S. chamaejasme* in the mountain system by comparing the elevational patterns of phenological matching between *S. chamaejasme* and its potential pollinators across different years with varying temperature fluctuations.

## 2 Materials and methods

### 2.1 Study area and focus species

The study site was located within the Zhengnangou watershed of the Qilian Mountains (38°33′20″-38°35′10″N, 100°8′47″-100°13′46″E), with an elevation range of 2400-3100 meters. The natural landscape within the watershed is representative of the region. Based on monitoring data from the comprehensive observation station within the watershed (elevation: 2750 meters), the long-term average temperature was 1.7°C, and the annual precipitation ranges between 300-500 mm, indicative of a typical temperate continental climate (Du et al., 2019). Grasslands cover 59.4% of the watershed area, making it the most widespread vegetation type (Wang et al., 2022). The grasslands are species-rich, including life forms such as grasses, sedges, and forbs (Wang et al., 2022). Dominant species include *Stellera chamaejasme* L*., Stipa krylovii* Roshev*., Potentilla acaulis* L*., Thermopsis lanceolata* R. Br*., and Polygonum viviparum* L*.*, primarily distributed on sunny slopes, semi-sunny slopes, and semi-shady slopes (Wang et al., 2022).

*S. chamaejasme* is a perennial herbaceous plant and one of the primary poisonous weeds in the natural grasslands of northern and northwestern China (Sun et al., 2009). It exhibits strong reproductive capacity and is highly drought and cold tolerance (Sun et al., 2009). *S. chamaejasme* has hermaphroditic flowers, with capitula borne at the apex, typically consisting of 5 to 15 inflorescences per individual and a white, radially symmetric corolla, and being self-incompatible, it relies on insect-mediated pollination and seed reproduction, with a relatively high seed set rate (Zhang et al., 2011). Typically, in the Qilian Mountains, *S. chamaejasme* resumes green growth in early May, flowers from mid to late June, sets fruit in July, and completes its life cycle by late summer (Gong and Huang, 2007). In recent years, the spread of *S. chamaejasme* populations has become increasingly severe due to the impacts of climate warming and grazing disturbances, posing a continuous threat to the survival of high-quality forage species (Li et al., 2024).

### 2.2 Investigation and monitoring

#### 2.2.1 Investigation of flowering period

Sampling sites were established along an elevation gradient (at 100m intervals) within the distribution range of *S. chamaejasme*. The selection of sampling sites was based on criteria of large continuous grassland areas with the same slope direction and minimal human disturbance. Five sample points were set up between 2500m and 2900m. Within each sampling site, an area of 50m by 50m was designated, and within this area, four 2m by 10m transects were randomly placed. All *S. chamaejasme* individuals appearing within these transects over time were marked and monitored. Continuous phenological monitoring was conducted on 553 individuals of *S. chamaejasme* during their reproductive season.

The flowering period was monitored from May to August of 2021 and 2022, from when the first individual began to flower until the last individual finished flowering. Monitoring was conducted every 2 to 5 days under clear weather conditions, recording the flowering abundance of each individual during each session. Monitoring sessions at different altitudes were completed within the same day, with a total of 10 sessions conducted per year at each altitude. At each sampling site, an air temperature and humidity recorder (MicroLite 5032, Israel) and a tip-bucket rain gauge (QT-50, China) were installed to simultaneously record temperature and precipitation at different altitudes, with meteorological data collected every half-hour.

#### 2.2.2 Monitoring of insect visitation

Current methods for monitoring insect visitation to flowers include timing with a stopwatch, direct observation (stationary and tracking), and recording with digital or video cameras (Gong and Huang, 2007; Gilpin et al., 2017). Compared to traditional methods, high-definition cameras provide more precise timing through video playback and allow for larger sample sizes (Westphal et al., 2008; Gilpin et al., 2017). Since *S. chamaejasme* has numerous capitula, attracts various flower-visiting insects. We chose to use high-definition cameras to record insect visitation behaviors, ensuring accurate species identification, potential pollinator determination, and documentation of the visitation process, thus ensuring data continuity and accuracy.

Flower visitors were monitored on clear days (with daily maximum temperatures above 15°C) during frequent insect activity hours from 9:00 to 18:00, starting from the beginning of the flowering period of individual plants. A high-definition digital camera (Sony, Japan, 48-megapixel) was used to continuously record the flower-visiting behavior of insects on the open capitula. The selection of individuals for filming was based on those with 8-10 branches per individual (with 8-10 fully blooming capitula per branch). The number of cameras deployed at each altitude was proportional to the abundance of flowering individuals, with at least two cameras used for replication in each monitoring session at each monitoring site. Throughout the monitoring period, a total of 90 cameras were deployed, capturing corresponding flower visit records. During post-processing, video playback was used to extract information on the identity of visiting insects, the frequency of visits for each insect, and the duration of each visit.

To study the population dynamics of flower-visiting insects, Malaise traps were employed to collect insect individuals. Beginning at the start of the flowering period, Malaise traps were placed at the center of each 50m by 50m sampling site, maintaining a certain distance from the transects. The traps were set every three days for a two-day capture period, after which they were removed to allow insect populations to recover naturally. This method ensured continuous population data while avoiding the impact of intensive collection on insect populations (O’Connor et al., 2019; Buffington et al., 2021). Since Malaise traps are less effective in capturing Coleoptera and Lepidoptera, yellow pan traps were also used to attract insects sensitive to colors by filling the pans with a small amount of alcohol (Buffington et al., 2021). We used three yellow pan traps at each sampling site, placing them 5 meters away from the Malaise trap, with each pan trap positioned at a 120-degree angle around the Malaise trap. This arrangement was consistent across all altitudes. The placement and retrieval frequency of the yellow pan traps matched that of the Malaise traps. Throughout the monitoring period, the Malaise traps and yellow pan traps were deployed and retrieved 19 times, with a total deployment duration of 38 days. The captured insects were counted and preserved as specimens for species identification.

### 2.3 Data analysis

#### 2.3.1 Determination of the flowering phenology

The flowering abundance data of *S. chamaejasme* were standardized by calculating the ratio of the flower abundance on each monitoring date to the total flower abundance across all monitoring dates. The standardized time series curves were then smoothed and fitted using the Gumbel Probability Density Function, which is widely used for probability analysis of extreme values in ecology, hydrology, and meteorology (Katz et al., 2005; García and Borda-de-Água, 2017), expressed as


$$y=y_{0}+Aexp\left\{-exp\left[-\left(\frac{x-x_{0}}{\omega }\right)\right]-\left(\frac{x-x_{0}}{\omega }\right)+1\right\}$$

where *x* and *y* represent time and standardized flower abundance, respectively, *y_0_* is the minimum value of *y* (set to 0 in this study), *A* is the amplitude between *y_0_* and the highest point of the curve, *x_0_* is the time corresponding to the highest point of the curve (i.e., the center of the curve, as the location parameter), and *ω* is the width of the curve (as the scale parameter). The significance of the fitting was tested using the *F*-test with a significance level of *p* < 0.05. Additionally, the mean goodness-of-fit (*R*²) of the data exceeded 0.95, indicating that the fitted curves effectively represented the distribution characteristics of the observed values. For the fitted flower abundance curves, the time corresponding to the peak (*x_0_*) was designated as the full bloom period (i.e., the peak of the flowering period). The start and end of the flowering period were determined using the relative threshold method, identifying the dates on the ascending (left) and descending (right) curves that reached 5% of the peak value as the start and end of the flowering period, respectively. The difference between these two dates was defined as the length of the flowering period (d). To examine the differences in flowering phenology of *S. chamaejasme* at different altitudes, Tukey’s Honest Significant Difference (HSD) test was conducted in the analysis of variance (ANOVA). The changes in the start and end times of the flowering period along the altitude in the same year were analyzed using general linear regression.

#### 2.3.2 Determination of major potential pollinators

For the flower visitation videos we collected, an initial manual review categorized behaviors into “brief pauses” (visits under 5 seconds), “prolonged resting” (visits over 60 seconds), and “petal nibbling.” Videos were selected based on whether the insect made contact with the anthers and stigma (Boff et al., 2018), determining their validity, and documenting the duration and frequency of visits (Ojija et al., 2019). From a total of 373 videos, 191 were ultimately deemed valid. Taxonomic experts identified insect species using both video images and specimens of the same insects captured in the videos. Prior research indicated considerable differences in pollination characteristics between insect families, but smaller differences within families; therefore, families were used as the functional classification basis to determine the flower-visiting insect community of *S. chamaejasme* (Aleixo et al., 2017). In practice, more insect groups were captured through traps than those appearing in the videos of *Stellera chamaejasme*. To maintain consistency, the captured insects were screened according to the morphological characteristics of those appearing in the videos. Since *S. chamaejasme* is a generalized pollinator plant attracting various insects, the pollination contribution of each potential pollinator was determined by the visitation frequency and duration for each family (Gong and Huang, 2007; Boff et al., 2018), weighted equally at 50% of each (visiting rate (%) = frequency of visits per family/total frequency of all families at all altitudes * 50% + duration of visits per family/Total duration of all families at all altitudes * 50%).

Results were further standardized to the inflorescence level to account for the potential effects of differences in the number of inflorescences between *S. chamaejasme* individuals at different elevations on visiting rate comparisons. For visit duration, records of visits lasting less than 5 seconds were first removed to exclude short stays by insects. Then the total duration was ratioed based on the number of inflorescences of the monitored individuals. Finally, records of single inflorescence visits lasting more than 60 seconds were removed to exclude the effects of prolonged resting on the analysis results. For visit frequency, effective visits were counted, assuming that each insect visit included all inflorescences on the individual plant. The average visiting rate over two years was used to determine the pollination contribution of each flower-visiting insect. Due to the similar pollination contribution of insects, it was finally determined that insects with visiting rates exceeding 10% were the major potential pollinators. The major potential pollinators mean that they are the most persistent and active visitors of *S. chamaejasme*.

#### 2.3.3 Determination of insect foraging

Insects collected using Malaise traps and pan traps were categorized by family, based on the major potential pollinator groups identified from flower-visitation videos. These insect counts were then standardized using a method similar to that applied to plant flower abundance data. Specifically, the number of insects on each monitoring date was expressed as a ratio of the total number of insects of the same family across all monitoring dates. The standardized abundance curves were smoothed and fitted for each potential pollinator group using the Gumbel probability density function (Katz et al., 2005; García and Borda‐de‐Água, 2017). Since insect activity throughout the year can be influenced by multiple generations, nectar availability, predator dynamics, and adverse weather conditions, their abundance curves may exhibit multiple temporal peaks rather than a single peak distribution (Thomson and Page, 2020). Therefore, we applied a multi-peak fitting method to smooth the standardized data, identifying the corresponding time points for multiple peak values.

#### 2.3.4 Phenological matching analysis

Given the reliability of the phenological peak in the trend test and its evolutionary significance, we used the peak time difference to measure the absolute mismatch degree of phenological matching between *S. chamaejasme* and its major potential pollinators (Miller-Rushing et al., 2008). A larger peak time difference indicates poorer phenological matching, i.e., a higher degree of phenological mismatch, whereas a smaller difference suggests better matching or lower mismatch. Before analysis, we needed to accurately determine the phenological peak periods of the major potential pollinators. To achieve this, we synthesized the abundance dynamics information of the major potential pollinators by weighting their temporal abundance according to their visitation rates, resulting in a composite abundance dynamic curve. Specifically, for each insect group, we first standardized the abundance data over time by calculating the ratio of the insect count on each monitoring date to the total count of the same family across all monitoring dates. Then, we multiplied these standardized temporal abundance data by their respective visitation rate proportions to obtain weighted standardized temporal abundances for each group. Finally, we summed the weighted abundance data of the four groups (*Meloidae*, *Tachinidae*, *Scarabaeidae*, and *Noctuidae*) and fitted them with a Gumbel probability density function to smooth the curve, ultimately identifying the activity peak periods of the major potential pollinators. Considering the possibility of multiple peaks in the temporal distribution of insect activity, the peak periods of insect abundance closest to the flowering peak were used to calculate the peak time difference for phenological matching.

To analyze the elevation differences in phenological matching between *S. chamaejasme* and its major potential pollinators, we established a linear regression relationship between the peak time difference and elevation, which was analyzed separately for each year. This approach allowed us to explore the sensitivity of phenological matching to elevation differences by examining the slope of the fitted linear relationship. Given that *S. chamaejasme* completes its flowering period before the end of July across all elevations, and considering the thermal accumulation and vernalization required for flowering, we defined the period from January to July as the effective timeframe for assessing the temperature impact on flowering. We calculated the average temperature during this period and used it as the independent variable. Then, we used the peak time difference as the dependent variable to analyze how sensitive phenological synchrony is to temperature variations through linear regression and analyzed each year separately. The slope of this regression represented the temperature sensitivity of phenological matching. Additionally, we considered interannual differences by comparing the trends of phenological matching across elevations under varying temperature fluctuations in different monitoring years (2021 and 2022). This comparison allowed us to explore the impact of varying environmental temperature conditions on the sensitivity of phenological matching to elevation and temperature between the two years.

## 3 Results

### 3.1 Phenological patterns of *S. chamaejasme* flowering

In the study area, early-season (January to May) precipitation in 2022 was 6.1% lower compared to 2021, and the accumulation of low temperatures (daily mean temperatures of 0-5°C) before the season (January to May) saw a 9.7% decline. Conversely, there was a 34.2% rise in heat accumulation (the cumulative sum of daily mean temperatures greater than 0°C) before the flowering period (January to May), with high temperatures continuing throughout this period (early June to late July). This created a relatively warmer growth environment for *S. chamaejasme* populations at all elevations, accompanied by a significant temperature gradient, with a lapse rate of 0.69°C for every 100 meters of elevation gain. Moreover, the average annual temperature rise of 0.56°C, is markedly higher than the interannual temperature rise of 0.02°C per year observed over the past 58 years, indicating notable temperature fluctuations between the study years. Monitoring the phenological patterns of *S. chamaejasme* flowering across various elevations showed consistent elevation-related trends between the study years. Both the onset and end of the flowering period started later with increasing elevation, with average shifts of 7.0 days per 100m for the onset (*R²* = 0.80, *p* < 0.001) and 6.5 days per 100m for the end of flowering (*R²* = 0.78, *p*< 0.001), calculated using general linear regression.

**Figure 1:**
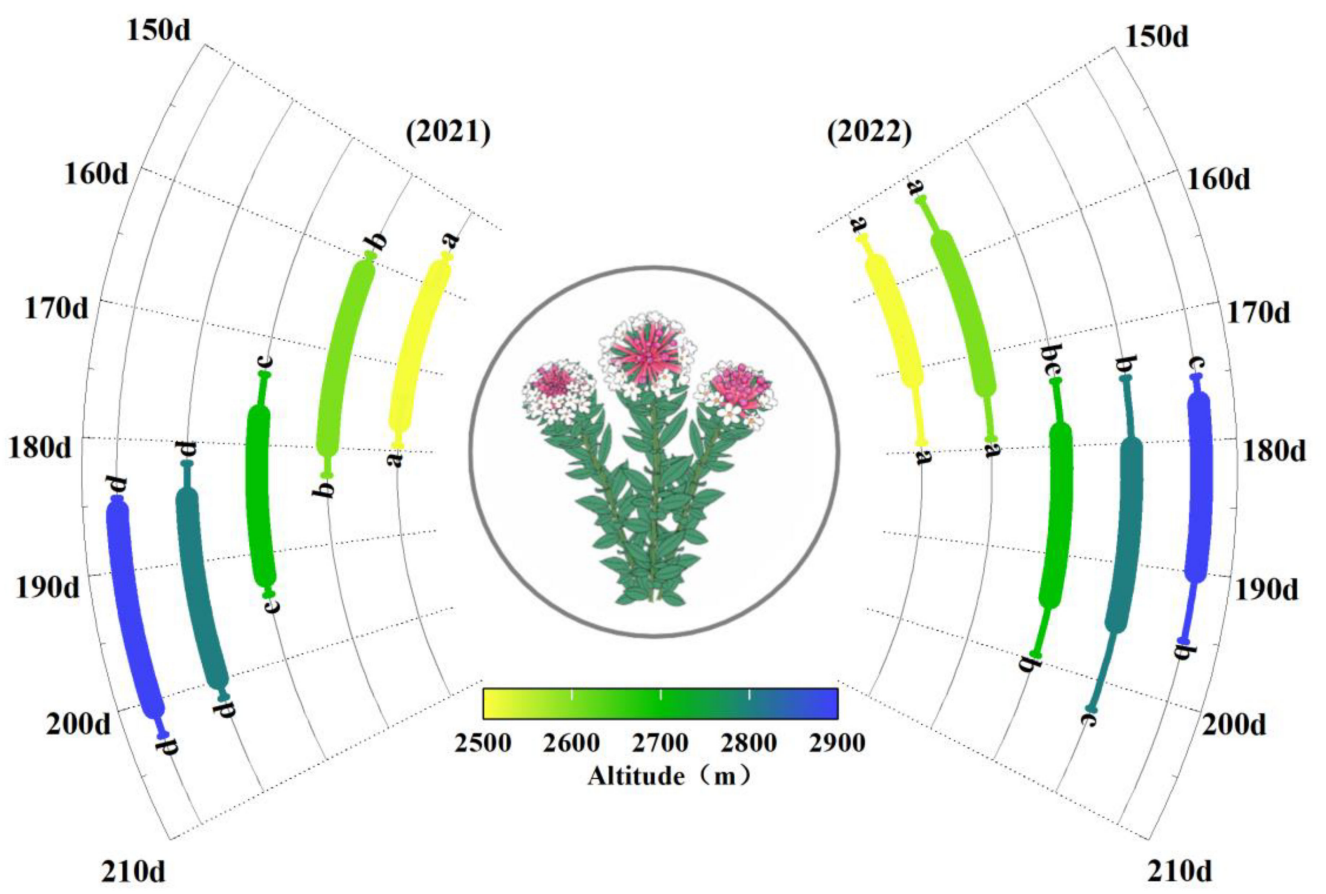
Altitudinal patterns and interannual variation in the phenology of *S. chamaejasme*. According to the ANOVA test, different letters represent the same phenological event (the start/end of the flowering period) in the same year with significant differences among different elevations.

Additionally, temperature variations between the two years led to differences in the flowering period of *S. chamaejasme*. Overall, in 2022, the onset and end of the flowering period were on average 3.2 days and 3.7 days earlier, respectively, compared to 2021(**Figure 1**, **Supplementary Table S1**). This interannual phenological difference also exhibited elevation specificity, with the most pronounced changes occurring at higher elevations. For instance, at 2900m, the onset of flowering in 2022 was 8.3 days earlier than in 2021, whereas at 2500m, it was only 1.1 days earlier (**Figure 1**, **Supplementary Table S1**). In terms of the flowering duration of different years, there were marginally significant differences in flowering period length across altitudes in 2021 (*F*=2.83, *p*=0.041, *η*²=0.27), while in 2022, there were no significant differences (*F*=1.50, *p*=0.20, *η²=*0.019).

### 3.2 Visitation rates and abundance dynamics of major potential pollinators

**Figure 2:**
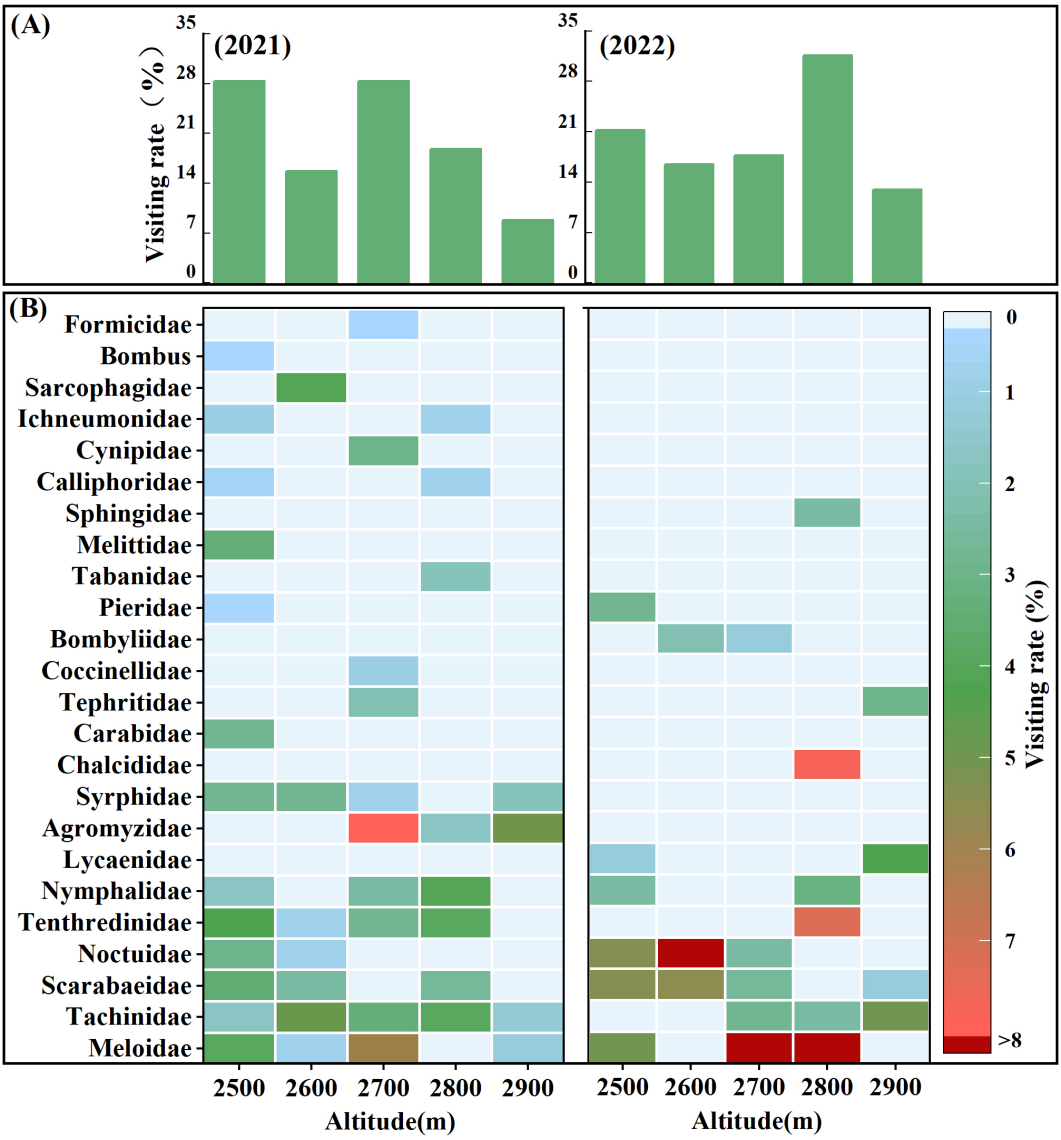
Altitudinal differences and interannual variation in visitation rates of flower-visiting insects to *S. chamaejasme*. The total visitation rate **(A)** for all insects at each altitude in 2021 and 2022, along with the corresponding visitation rate **(B)** for each insect family, is represented with filled colors indicating the intensity of the visitation rate. The legend indicates the intensity of specific visit rates.

*S. chamaejasme* attracted a diverse assemblage of insect visitors, spanning 24 families across 4 orders (**Figure 2**, **Supplementary Tables S2, S3**). Considering both visitation frequency and duration across the entire altitudinal range in the two years, the major potential pollinators of *S. chamaejasme* with a high two-year average visiting rates (%) were identified as *Meloidae* (17.3%), *Tachinidae* (12.1%), *Scarabaeidae* (11.2%), and *Noctuidae* (10.0%). Other insects were categorized as unstable visitors or occasional visitors with disproportionate visitation frequency and duration. Although many species of flower-visiting insects were present, there was substantial variation in visitation rates across different elevations and between years (**Figure 2**, **Supplementary Tables S2, S3**). From 2021 to 2022, the number of visiting insect species mostly decreased, with an average reduction of 1.6 families per 100 m (*R^2^* = 0.84, *F* = 21.33, *p* = 0.019), while the overall visiting rate did not change significantly. Although the total visitation rate did not change significantly, there were notable variations within specific elevation ranges: between 2500m and 2700m, the visitation rate decreased by an average of 5.7%, whereas between 2800m and 2900m, it increased by an average of 8.5%. The number of visitor families at all altitudes in 2021 (20 families) was greater than in 2022 (12 families), with only 8 families common to both years. The interannual differences in major potential pollinators’ composition were mainly due to variations in the unstable and occasional visitors, while the overall visitation by major potential pollinators remained relatively stable between the years.

**Figure 3:**
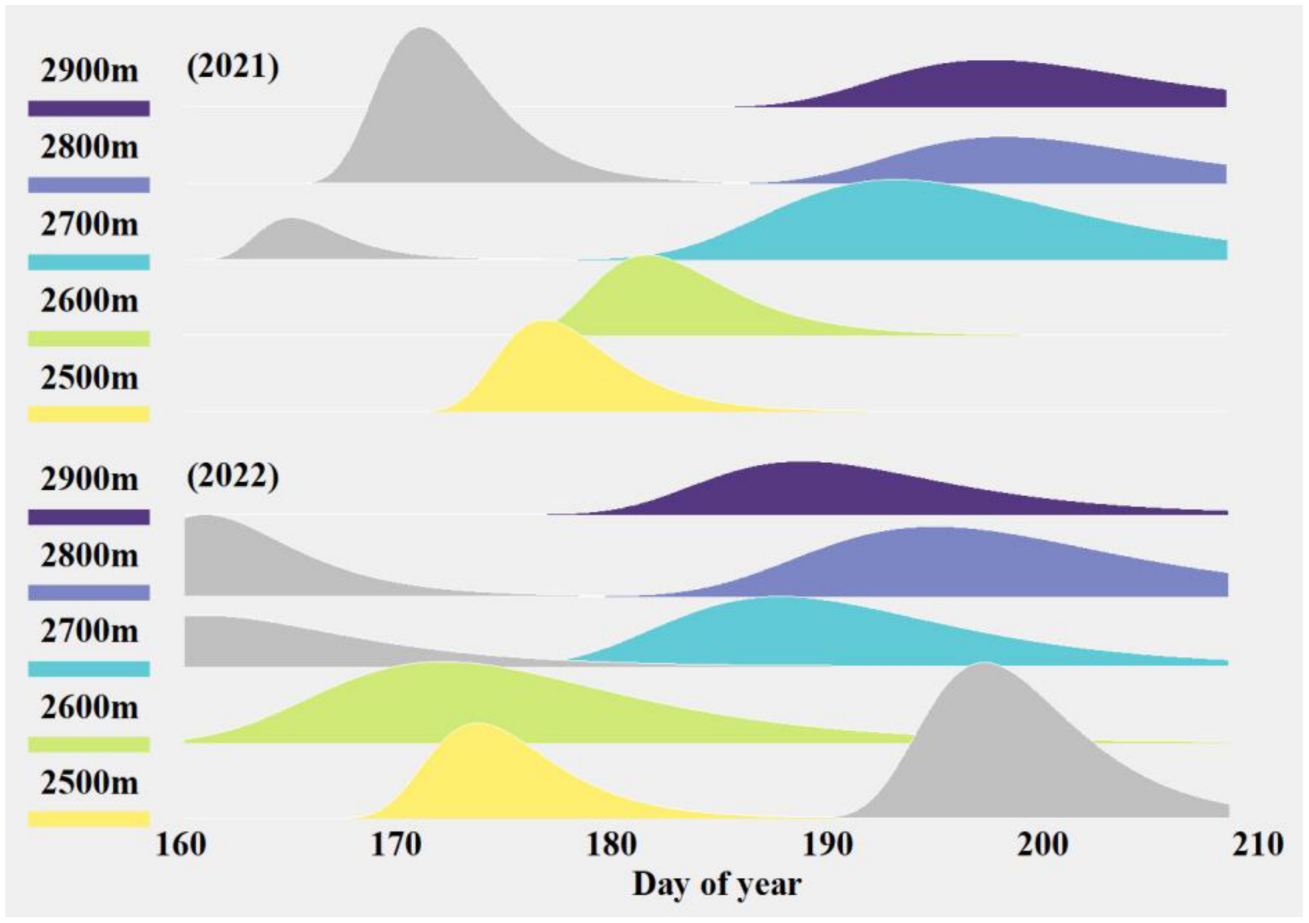
The combined abundance dynamics of the four major potential pollinators in 2021 and 2022. Different colors represent the phenology of pollinator activity at different altitudes. Where certain altitudes exhibited two peaks of flower-visiting activity in the main pollinators, the pollinator peak closest to *S. chamaejasme* bloom is shown in color while the peak furthest away from *S. chamaejasme* bloom is shown in grey.

The standardized abundance over time of major potential pollinators of *S. chamaejasme*, weighted by their respective visitation rates, is shown in **Figure 3** and **Supplementary Table S4**. The abundance dynamics of the potential pollinators typically exhibited a multi-peaked pattern, usually bimodal, with two relative abundance peaks separated by a certain time interval (usually more than half a month). This pattern likely reflects temporal distribution differences among various potential pollinators. Additionally, there were elevation-specific differences in the activity patterns of insects, with foraging peaks rarely occurring simultaneously across elevations. From the perspective of inter-annual changes, the timing of peak insect abundance at the same elevation varied between years. In 2022, the peak abundance periods were generally earlier than 2021, with an average advancement of 5.8 days.

### 3.3 Elevation differences in phenological matching

**Figure 4:**
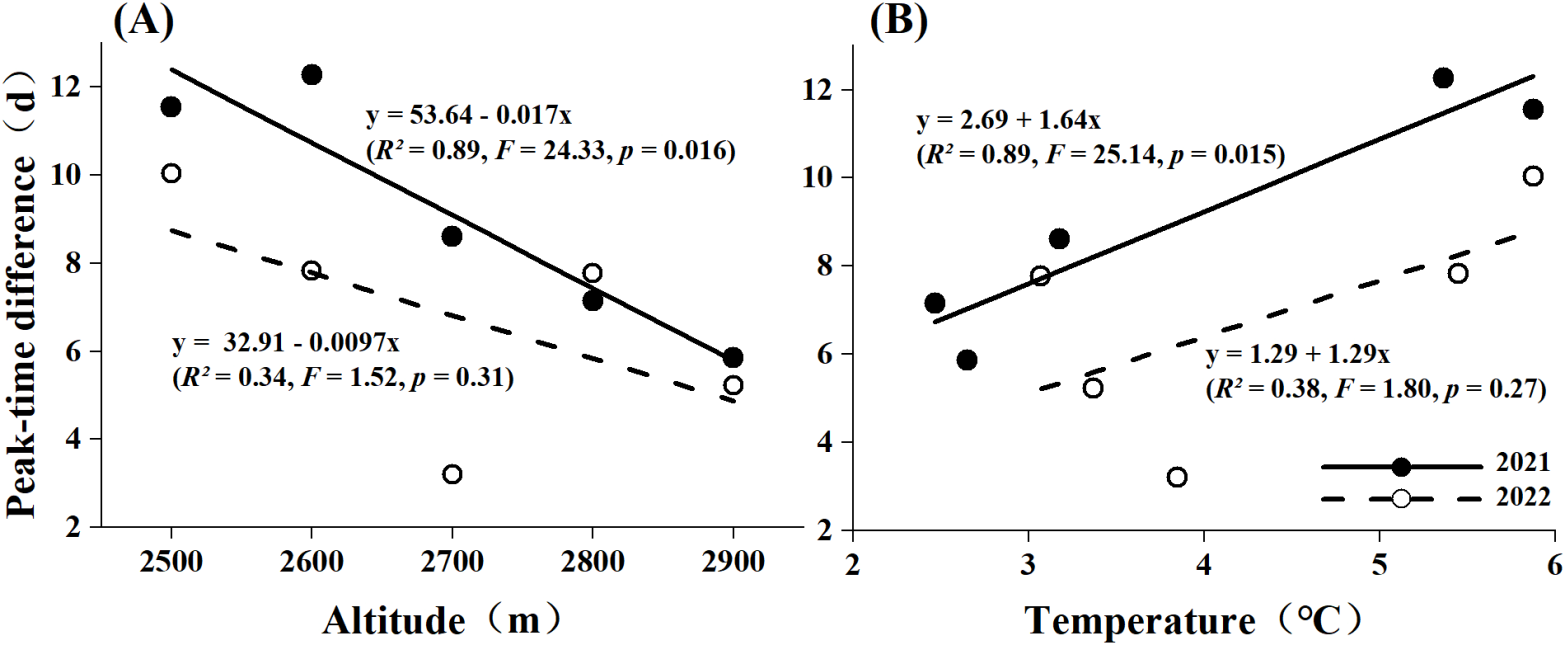
Phenological matching under altitudinal **(A)** and average temperature **(B)** differences in 2021 and 2022. The straight line represents the linear fitting curve of the peak time difference with altitude **(A)** and its corresponding average temperature **(B)**, based on the linear regression model of the form y = β_0_ + β_1_x. The solid line is the fitting curve of 2021, and the dashed line is the fitting curve of 2022.

By comparing the peak flowering periods of *S. chamaejasme* with the peak abundance periods of neighboring pollinators across different elevations and varying temperatures, we assessed the phenological matching between them. **Figure 4** and **Supplementary Tables S5, S6** show a certain degree of phenological mismatch between *S. chamaejasme* and its major potential pollinators at all elevations. This mismatch is characterized by the nearest activity of major potential pollinators occurring later than the peak flowering abundance (Δ2021 = 9.1 days, Δ2022 = 6.8 days). Linear regression fitting indicates that phenological matching is greater at higher elevations compared to lower elevations, and this trend reached statistical significance in 2021 (*F* = 24.33, *p* = 0.016). Between the two years, the mismatch between the peak flowering period of *S. chamaejasme* and the peak activity period of its major potential pollinators shorted in 2022 compared to 2021, with an average reduction of 2.3 days. This reduction in mismatch was more pronounced at lower elevations. For example, the phenological matching at low and middle elevations improved by an average of 3.8 days, whereas at elevation of 2900m, less than a day in advance. When correlating elevation with temperature, we found that the peak time difference showed a positive correlation with temperature (*β*_1_ = 1.64, **Figure 4**, **Supplementary Table S5**). However, in 2022, the trend of increasing peak time difference with temperature slowed, as reflected by the decrease in the regression coefficients (*β*_1_ = 1.29, **Figure 4**, **Supplementary Table S6**).

## 4 Discussion

### 4.1 Phenology of *S. chamaejasme* flowering period

Flowering phenology is a crucial rhythmic event in the sexual reproduction process of angiosperms, which largely determines the reproductive behavior of plants and is regarded as a key fitness influencing factor for many flowering plants (Kudo and Cooper, 2019). Studies have revealed that the flowering phenology of early-flowering plants is highly sensitive to environmental changes, similar to other vegetative growth stages like budburst and greening (Muhanguzi and Ipulet, 2012). In the studied high-altitude grassland communities, *S. chamaejasme* begins flowering in early June, classifying it as an early-flowering species within the region’s flowering period of June to August (Wang et al., 2024). Previous research has shown that species that flower earlier in the spring are more sensitive to temperature changes (Bertin, 2008; Wolkovich et al., 2012). This characteristic was also confirmed by our comparisons of the years 2021 and 2022 where the flowering period began earlier in the warmer year (2022), especially in high-altitude populations, possibly suggesting that *S. chamaejasme* is actively adapting to variations in environmental temperature.

Plants may adapt to environmental changes through phenological plasticity, such as extending flowering periods or advancing flowering onset, thereby increasing reproductive opportunities (Wang et al., 2024). The high sensitivity of the flowering period to temperature may be a favorable condition for its rapid expansion in high-altitude areas. Studies have shown that with rising environmental temperatures, pollinator activity may advance more significantly than changes in plant flowering periods (Kudo and Ida, 2013). In this case, for plants such as *S. chamaejasme*, the high sensitivity of their flowering phenology to environmental changes will help maintain synchronization with pollinators to track environmental signals, sustaining high reproductive advantages in natural communities and promoting population expansion.

### 4.2 Major potential pollinators of *S. chamaejasme*

For entomophilous, seed-reproducing angiosperms, pollinators play a crucial role in the pollination process (Ollerton, 2017). Our research identifies *S. chamaejasme* as a generalist pollinator type, visited by insects from 24 families across *Coleoptera*, *Diptera*, *Lepidoptera*, and *Hymenoptera*. Compared to other studies, we observed a greater variety of flower-visiting insects, providing more comprehensive information on potential pollinators, as our monitoring encompassed the full altitudinal range of *S. chamaejasme* distribution, offering a more objective perspective than single-point or single-altitude studies (Sun, 2022). We also noted that in a warmer year (2022), the number of visitor groups generally decreased.

Although *S. chamaejasme* has many potential pollinators, the visitation rates of different insects vary significantly with altitude and year, particularly among unstable visitors and occasional visitors with generalized pollination attributes. These insects are likely to switch to other more “valuable” plant hosts if their activity period decouples from the flowering period (Wratten et al., 2012). For *S. chamaejasme*, interactions with these insects are more opportunistic, constantly changing with interannual temperature fluctuations or altitudinal environmental differences. Conversely, the major potential pollinators of *S. chamaejasme* (*Meloidae*, *Tachinidae*, *Scarabaeidae*, and *Noctuidae*) exhibit relatively stable visitation rates across the years, establishing stable interaction relationships.

Our study focuses on major potential pollinators rather than the most frequent flower visitors because we lack data on the pollination efficiency (i.e., pollen transfer rate) of each group. Instead, we use visitation rates to identify potential pollinators (Boff et al., 2018). For a more objective analysis, we conservatively define major potential pollinators. Our temporal monitoring of the population dynamics of the four major potential pollinators shows that insect abundance curves often exhibit multi-peak distribution patterns. This phenomenon may result from inconsistent feeding activity times among different insect groups (Thomson and Page, 2020). Additionally, even within the same insect group, individual abundance dynamics often do not follow a single-peak distribution, possibly influenced by multiple generational reproduction, predator dynamics, and adverse weather events (Stefanescu et al., 2013). Although we have gained further insights into the major potential pollinators of *S. chamaejasme*, it is important to acknowledge that classifying at the family level remains a relatively coarse standard. Future studies with detailed classification at the genus and species levels would provide more precise data on pollinators, though this approach would be challenging.

### 4.3 Phenological matching between *S. chamaejasme* and its potential pollinators

Pollinating insects form specific coupling relationships with flowering plants during their visitation periods, shaping the phenological matching patterns between plants and pollinators (Hegland et al., 2009). The establishment of phenological matching is crucial for the reproductive success of flowering plants and depends on the synchronization between the plant flowering phenology and the activity rhythms of their pollinators (Root et al., 2003). Previous studies have found that the phenological matching between plants and pollinators is highly susceptible to environmental changes, leading to asynchronous shifts in their phenological periods and resulting in phenological mismatches (Rafferty and Ives, 2012). In our study, we observed a general phenological mismatch between the peak flowering period of *S. chamaejasme* and the peak abundance of its major potential pollinators, which is consistent with previous research suggesting that achieving perfect phenological matching is uncommon in natural communities (Rafferty and Ives, 2011; Forrest, 2015; Wang et al., 2024). However, when comparing the phenological matching across different altitudinal zones, we observed that high-altitude regions exhibited a greater degree of phenological synchrony compared to low-altitude regions. This suggests that at higher altitudes *S. chamaejasme* populations experience higher phenological synchrony with their major potential pollinators, possibly stabilizing reproductive capacity and demographics. This phenomenon might be a significant driving factor behind the rapid upward invasion of *S. chamaejasme* in mountainous systems, as reported in recent studies (Wang et al., 2019; Zhang et al., 2015).

Temperature plays a crucial regulatory role in the life cycle activities of both plants and animals (Bartomeus et al., 2011; Kehrberger and Holzschuh, 2019). Consequently, temperature changes associated with climate change significantly impact the phenological matching between plants and pollinators (Bartomeus et al., 2011; Rafferty and Ives, 2011). Through interannual comparisons of the phenological matching between *S. chamaejasme* and its major potential pollinators, we found that in relatively a warmer year (e.g., 2022), the degree of phenological mismatch decreased. This might be due to differential responses of flowering phenology and insect activity to rising environmental temperatures. While both the flowering period of *S. chamaejasme* and the activity period of its major potential pollinators started earlier at higher temperatures in 2022, the degree of advancement was not the same, narrowing the gap between their peak times. The increased phenological matching may suggest that the higher environmental temperatures in 2022 facilitated more synchronized interactions between *S. chamaejasme* and its major potential pollinator within its distribution range. Given that *S. chamaejasme* does not have the ability for vegetative reproduction (Xing et al., 2004) and relies entirely on pollinators for cross-fertilization, the increase in phenological synchrony may be one of the key factors influencing its successful establishment and spread.

When comparing the changes in phenological matching across different altitudes in a warmer year (2022), we observed that the increase in phenological matching was more pronounced at lower altitudes compared to higher altitudes. This could be attributed to the fact that the peak flowering period of *S. chamaejasme* typically occurs earlier than the insect activity peak, especially at higher altitudes, where flowering time advances more significantly, maintaining relative synchronization with insect activity. In contrast, at lower altitudes, the smaller shift in flowering time, compared to the more substantial advancement in insect activity, narrows the peak time gap between them. This suggests that rising temperatures may strengthen the interaction between low-altitude populations and their major pollinators, gradually reducing the altitude and temperature sensitivity of phenological matching. We speculate that the current increase in environmental temperatures could add to the continued success of *S. chamaejasme* populations expanding across various altitudes in mountainous systems. However, whether this phenological matching will further strengthen in the future remains to be seen and would require long-term monitoring.

It is important to note that our analysis primarily focuses on phenological peaks, which may not capture the full extent of phenological overlap outside of the peak periods. Many interactions between plants and pollinators could occur before or after the peak activity periods, particularly for taxa with extended activity durations. Furthermore, phenological activity is often asymmetrically distributed around the peak, and focusing solely on peak times may obscure important overlapping phases that contribute to overall phenological matching. While peak times are commonly used in phenological studies, future research could benefit from considering full phenological distributions to better understand the dynamics of plant-pollinator interactions across different environmental conditions. This is particularly crucial as environmental changes may alter the symmetry of these distributions, leading to more complex shifts in phenological matching.

## 5 Conclusion

This study provides a case demonstrating that fluctuations in environmental temperatures between 2021 and 2022 led to changes in the phenological matching between the flowering abundance of *S. chamaejasme* and the abundance of major potential pollinators across different altitudinal gradients in mountain systems. Our results indicate that short-term environmental warming has enhanced the phenological synchrony between the dominant poisonous weed *S. chamaejasme* and its major potential pollinators in the Qilian Mountains, with reduced temperature sensitivity observed at various altitudes. This synchrony may be one of the important factors facilitating the reproductive expansion of *S. chamaejasme* across different elevations. We propose that this mechanism could be a key driver behind the continued spread of insect-pollinated, seed-reproducing invasive plants. Our case study emphasizes the importance of focusing on phenological matching between plants and pollinators for accurate predictions of population dynamics under environmental change. As climate change intensifies, patterns of phenological matching may become crucial in determining species interactions. Mismatches in phenology often lead to reduced visitation by efficient pollinators, which can significantly influence species establishment and reproductive success in new environments. Therefore, understanding and predicting phenological synchronization is essential for managing and conserving biodiversity in the face of ongoing climate change.

## Data Availability

The raw data supporting the conclusions of this article will be made available by the authors, without undue reservation.
