# Supplementary material for: Interannual temperature rise leads to more uniform phenological matching between invasive Stellera chamaejasme and pollinators across elevations
Source: Front Plant Sci. 2024 Oct 11;15:1445083. doi: 10.3389/fpls.2024.1445083 (PMC11502355; doi:10.3389/fpls.2024.1445083)
Supplement: Supplementary file 1 [file DataSheet1.pdf]

Table S1 Flowering phenology of *S. chamaejasme* in 202 and 2022.

Year	Altitude (m)	First flowering date	SD	End flowering date	SD	Flowering duration	SD
2021	2500	158.000	2.000	176.500	2.828	18.500	3.586
	2600	161.200	1.789	179.800	3.033	18.600	3.912
	2700	177.250	3.808	192.125	1.727	14.875	3.137
	2800	184.625	2.875	199.875	1.642	15.250	2.252
	2900	185.429	0.976	200.571	2.149	15.143	1.676
2022	2500	156.946	3.652	172.982	5.848	16.036	4.279
	2600	157.553	4.821	173.447	5.454	15.894	4.653
	2700	178.721	4.805	194.081	5.354	15.360	5.649
	2800	180.319	5.773	197.221	5.226	16.901	4.921
	2900	177.096	1.944	192.308	2.970	15.212	3.308

Table S2 Flower visit rates (%) of insects at different altitudes in 2021.

	2500m	2600m	2700m	2800m	2900m
Meloidae	3.74	0.73	5.64	0.00	1.07
Tachinidae	1.68	4.66	3.26	3.65	1.25
Scarabaeidae	3.47	2.25	0.00	2.53	0.00
Noctuidae	2.84	0.67	0.00	0.00	0.00
Tenthredinidae	4.09	0.73	2.66	3.70	0.00
Nymphalidae	1.63	0.00	2.31	3.86	0.00
Lycaenidae	0.00	0.00	0.00	0.00	0.00
Agromyzidae	0.00	0.00	7.60	1.61	4.74
Syrphidae	2.69	2.75	0.79	0.00	1.75
Chalcididae	0.00	0.00	0.00	0.00	0.00
Carabidae	2.61	0.00	0.00	0.00	0.00
Tephritidae	0.00	0.00	1.99	0.00	0.00
Coccinellidae	0.00	0.00	0.94	0.00	0.00
Bombyliidae	0.00	0.00	0.00	0.00	0.00
Pieridae	0.47	0.00	0.00	0.00	0.00
Tabanidae	0.00	0.00	0.00	1.81	0.00
Melittidae	3.29	0.00	0.00	0.00	0.00
Sphingidae	0.00	0.00	0.00	0.00	0.00
Calliphoridae	0.54	0.00	0.00	0.83	0.00
Cynipidae	0.00	0.00	2.84	0.00	0.00
Ichneumonidae	0.83	0.00	0.00	0.81	0.00
Sarcophagidae	0.00	3.92	0.00	0.00	0.00
Bombus	0.45	0.00	0.00	0.00	0.00
Formicidae	0.00	0.00	0.32	0.00	0.00

Table S3 Flower visit rates (%) of insects at different altitudes in 2022.

	2500m	2600m	2700m	2800m	2900m
Meloidae	4.73	0.00	9.16	9.52	0.00
Tachinidae	0.00	0.00	2.65	2.34	4.75
Scarabaeidae	5.21	5.37	2.43	0.00	1.15
Noctuidae	5.07	9.06	2.36	0.00	0.00
Tenthredinidae	0.00	0.00	0.00	6.94	0.00
Nymphalidae	2.38	0.00	0.00	3.09	0.00
Lycaenidae	1.10	0.00	0.00	0.00	4.15
Agromyzidae	0.00	0.00	0.00	0.00	0.00
Syrphidae	0.00	0.00	0.00	0.00	0.00
Chalcididae	0.00	0.00	0.00	7.45	0.00
Carabidae	0.00	0.00	0.00	0.00	0.00
Tephritidae	0.00	0.00	0.00	0.00	2.91
Coccinellidae	0.00	0.00	0.00	0.00	0.00
Bombyliidae	0.00	2.05	1.14	0.00	0.00
Pieridae	2.76	0.00	0.00	0.00	0.00
Tabanidae	0.00	0.00	0.00	0.00	0.00
Melittidae	0.00	0.00	0.00	0.00	0.00
Sphingidae	0.00	0.00	0.00	2.25	0.00
Calliphoridae	0.00	0.00	0.00	0.00	0.00
Cynipidae	0.00	0.00	0.00	0.00	0.00
Ichneumonidae	0.00	0.00	0.00	0.00	0.00
Sarcophagidae	0.00	0.00	0.00	0.00	0.00
Bombus	0.00	0.00	0.00	0.00	0.00
Formicidae	0.00	0.00	0.00	0.00	0.00

Table S4 The peak periods (DOY) of insect abundance.

Altitudes(m)	2021(DOY)		2022(DOY)	
2500		176.68	173.60	197.12
2600		181.47	171.98	
2700	164.95	192.99	160.86	187.64
2800	171.00	198.02	161.00	194.83
2900		197.43		188.71

Table S5 Phenological matching under altitudinal and average temperature differences in 2021.

Altitudes(m)	Temperature(℃）	Peak-time difference(d)
2500	5.88	11.55
2600	5.37	12.27
2700	3.18	8.61
2800	2.47	7.15
2900	2.65	5.86

Table S6 Phenological matching under altitudinal and average temperature differences in 2022.

Altitudes(m)	Temperature(℃）	Peak-time difference(d)
2500	5.87	10.03
2600	5.45	7.82
2700	3.85	3.21
2800	3.07	7.77
2900	3.37	5.22
